# Effects of different exercise interventions on lower back pain: a systematic review and meta-analysis

**DOI:** 10.3389/fphys.2025.1694330

**Published:** 2026-01-06

**Authors:** Xingchi Liu, Wenbo Gao, Peirun Wu, Jiawang Huang, Jingjing Han, Xiuli Xu, Wanqiang Chen

**Affiliations:** 1 Department of Rehabilitation Medicine of the First Hospital of Lanzhou university, Lanzhou, Gansu, China; 2 The Second Clinical Medical School, Lanzhou University, Lanzhou, China

**Keywords:** low-back pain, exercise intervention, meta-analysis, rehabilitation, pain relief

## Abstract

**Objective:**

Lower back pain (LBP) is the leading cause of disability worldwide. This study evaluates the pain relief and functional benefits of exercise interventions for affected individuals to inform clinical practice.

**Methods:**

We searched nine electronic databases for randomized controlled trials (RCTs) that examined exercise interventions for LBP.

**Results:**

We included 35 RCTs (n = 2,132). Exercise interventions were categorized into eight types: Pilates, yoga, core training, tai chi, walking, stretching, cycling, and deep-water running. Compared to usual care or other types of pain management interventions, exercise interventions demonstrated a significant overall difference in reducing pain (SMD = −0.81, 95% CI −0.91, −0.72; 17.31, P < 0.001). Subgroup analysis revealed that tai chi (SMD = −0.95), walking (MD = −1.05), and Pilates (MD = −1.14) exhibited the most significant analgesic effects. Regarding functional disability improvement, assessment using the Oswestry Disability Index showed significant efficacy for walking (MD = −6.34, P < 0.001), Pilates (MD = −4.73, P < 0.0001), and yoga (MD = −3.41, P = 0.002). However, assessment using the Roland–Morris Disability Questionnaire (RMDQ) indicated that only Pilates resulted in significant improvement (MD = −2.34, P < 0.001).

**Conclusion:**

Pilates, yoga, and walking reduce pain and improve function in non-specific LBP. Tai chi and core-stability training also achieve significant analgesia. The evidence for stretching and cycling remains inconclusive.

**Systematic Review Registration:**

https://www.crd.york.ac.uk/PROSPERO/view/CRD420251047326, identifier CRD420251047326.

## Introduction

Lower back pain (LBP) has remained the leading cause of disability worldwide for more than three decades (Hartvigsen et al., 2018). According to the 2021 Global Burden of Disease Study, approximately 730 million people currently live with LBP, and this number is projected to exceed 840 million by 2050 (GBD 2021 Low Back Pain Collaborators, 2023). Chronic non-specific LBP (CNSLBP) accounts for more than 85% of cases, and its consequences extend beyond pain and functional impairment to encompass depression, anxiety, sleep disturbance, and diminished work capacity, thereby imposing significant socioeconomic costs on patients, families, and society (GBD 2019 Diseases and Injuries Collaborators, 2020). Despite advances in pharmacological, interventional, and surgical treatment, long-term outcomes remain modest, and such approaches may carry risks of adverse events or high financial burdens. Consequently, safe, cost-effective, and scalable treatment strategies are urgently needed (Oliveira et al., 2018).

Exercise therapy, owing to its non-invasive nature, accessibility, and wide-ranging health benefits, is consistently recommended as a first-line treatment for CNSLBP in major international guidelines (National Guideline C. National Institute for Health and Care Excellence: Guidelines, 2016; Qaseem et al., 2017).

Over the past two decades, a variety of exercise modalities have been investigated, including traditional approaches such as Pilates, yoga, and tai chi, as well as more contemporary forms such as core-stability training, aerobic walking, cycling, stretching, and deep-water running. However, the evidence base for these interventions has evolved unevenly. For example, a recent systematic review and meta-analysis concluded that Pilates provides clinically meaningful improvements in both pain and disability compared to minimal interventions (Patti et al., 2024), whereas yoga has also shown consistent, though more modest, benefits in pain reduction and functional outcomes. In contrast, evidence regarding tai chi and aquatic-based exercises remains sparse or inconclusive, and trials investigating aerobic walking or cycling have reported conflicting results (Fransen et al., 2015).

Previous syntheses have consistently confirmed that virtually any structured exercise attenuates pain and disability in adults with LBP (Hayden et al., 2021); nevertheless, the relative merit of competing protocols remains indeterminate. Similarly, Cochrane overviews comparing motor-control, resistance, Pilates, or yoga interventions (Vandestienne et al., 2022; Cai et al., 2022) report overlapping 95% confidence intervals for pain intensity and function but provide no hierarchy of benefit. Consequently, current guidelines (Tomita et al., 2022) issue generic “remain active” recommendations, leaving clinicians without an evidence-based algorithm to match exercise type to patient phenotype. A quantitative comparative synthesis that integrates both direct and indirect randomized evidence is therefore urgently required to clarify which movement strategy, if any, optimizes clinically relevant outcomes in adults with chronic LBP.

Given these limitations, there remains a critical need for an updated and comprehensive synthesis of the comparative efficacy of different exercise modalities. To address this gap, we conducted a systematic review and network meta-analysis of randomized controlled trials (RCTs) to evaluate and rank nine mainstream exercise interventions for pain and disability outcomes in adults with CNSLBP. Our aim is to provide clinicians with high-quality evidence to guide individualized, evidence-based exercise prescriptions for this prevalent and burdensome condition (Qaseem et al., 2017; Fernández-Rodríguez et al., 2022; Shiri et al., 2018).

## Methods

### Protocol and registration

The protocol was prospectively registered with the International Prospective Register of Systematic Reviews (PROSPERO) under ID CRD420251047326. The review was conducted in accordance with the Preferred Reporting Items for Systematic Reviews and Meta-Analyses (PRISMA) statement (Page et al., 2021) and the *Cochrane Handbook*.

### Inclusion and exclusion criteria

Inclusion criteria comprised the following: (1) Population (P): adults (aged ≥18 and ≤80 years) in the general population diagnosed by a physician or rehabilitation specialist with CNSLBP (lasting ≥12 weeks), without concomitant organic lumbar spine pathology (infection, tumor, fracture, inflammatory spondyloarthritis, cauda equina syndrome, or radicular compression requiring surgical intervention); (2) Interventions (I): a course lasting at least 2 weeks and including at least six supervised or prescribed training sessions encompassing Pilates, yoga, core-stability training, tai chi, walking, stretching, cycling, or deep-water running. (3) Comparisons (C): usual care or other types of pain management interventions. (4) Study design: randomized controlled trials (RCTs). (5) Outcomes (O): pain intensity is assessed using the Visual Analog Scale (VAS) or a 0–10 numerical rating scale (NRS). Functional status is evaluated using the Roland–Morris Disability Questionnaire (RMDQ) or the Oswestry Disability Index (ODI). (6) Language: articles written in English, Chinese, Spanish, French, German, or Portuguese.

Exclusion criteria comprised the following: (1) non-RCT studies; (2) studies with inaccessible full texts; (3) conference abstracts, reviews, animal experiments, or duplicate publications.

### Literature search

**FIGURE 1:**
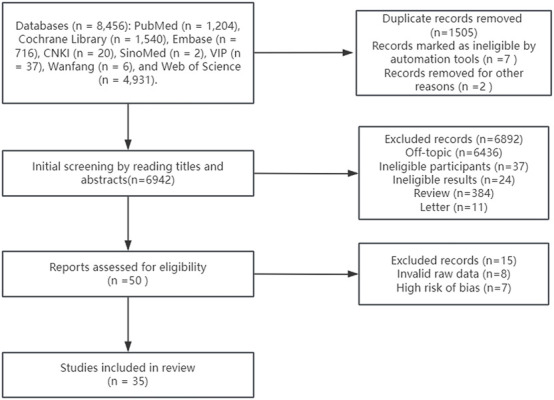
Flowchart of study selection and results.

A comprehensive search was performed across nine Chinese and English databases: CNKI, VIP, Wanfang Data Knowledge Service Platform, SINOMED, PubMed, Web of Science, CINAHL, Cochrane Library, and Embase. The search strategy combined subject terms and free-text words to optimize retrieval efficiency, including keywords such as “walking,” “yoga,” “tai chi,” “Pilates,” “lower back pain,” “core strengthening,” and “deep-water running.” The search timeframe was from the inception of each database to May 2025. Additionally, references of included studies were supplemented for retrieval. The search process is illustrated in Figure 1.

### Quality assessment of literature

The Cochrane Risk of Bias Tool (Version 6.0) was used to evaluate the quality of the RCTs, covering domains including random sequence generation, allocation concealment, blinding, completeness of outcome data, selective reporting, and other sources of bias. Each domain was carefully assessed as “high risk of bias,” “some concerns,” or “low risk of bias” based on predefined criteria. Two reviewers independently evaluated the risk of bias for each study; any discrepancies were resolved through discussion with a third reviewer.

### Data extraction

Two reviewers (GWB and WPR) independently extracted data from every included RCT. Disagreements were first resolved by re-checking the original publication and, if necessary, adjudication by a third reviewer. Extracted variables comprised bibliographic details, participant characteristics, sample size, mean age, intervention components (type, frequency, intensity, duration, delivery mode, and interveners), follow-up length, outcome measures (VAS, NRS, ODI, RMDQ, etc.), and key results. When primary outcome data (means, standard deviations, or event counts) were missing or presented only in graphs, we attempted to contact the corresponding author by e-mail (up to two reminders at 2 week intervals). Where no response was received, we used established statistical methods (Hozo (2005) for medians/ranges; Wan (2014) for inter-quartile ranges) to estimate missing statistics; all imputations are flagged in the evidence tables and sensitivity analyses were performed to assess their impact.

### Data synthesis and analysis

All eligible studies are summarized in Table 1. For analysis, data extracted from included publications were imported into Review Manager (RevMan) 5.4 software. Heterogeneity was assessed using the I^2^ statistic, where values of 25%, 50%, and 75% indicated low, moderate, and high heterogeneity, respectively. A random-effects model was used for data with high heterogeneity; otherwise, a fixed-effects model was applied. For the effects of different exercises on pain and dysfunction, weighted mean differences (WMD) were calculated if outcomes were measured using the same scales or indicators. If different scales or indicators were used across trials, standardized mean differences (SMD) with corresponding 95% confidence intervals (CI) were applied. Subgroup analyses were performed to explore potential sources of heterogeneity. In stratified meta-analyses, data from the literature were divided into subgroups based on intervention types (Pilates, yoga, core-stability exercises, tai chi, walking, stretching, cycling, and deep-water running). If the combined results showed high heterogeneity, the effect size and 95% CI of each study were reported with a narrative description. A P-value <0.05 was considered statistically significant. Funnel plots were used to examine the included literature to detect publication bias. Certainty of evidence was rated with the GRADE 4.0 approach. Each outcome started at “high” certainty and was downgraded for risk of bias, inconsistency, indirectness, imprecision, or publication bias. Final grades were “high,” “moderate,” “low,” or “very low”.

**TABLE 1:** Characteristics of included randomized controlled trials.

Author, year, country	Participant	Sample size (I/C)	Mean age, year	Intervention		Measure	Critical finding
				Intervention group (IG)	Control group (CG)		
Zuo et al. (2024) Pakistan	36 individuals with CNSLBP were recruited for the study from District Headquarters Hospital, Nankana Sahib	18/18	IG: 31 ± 5.44 CG: 35.77 ± 7.42	Retro-walking	Conventional treatment	NPRS ODI	This study showed a significant difference in the numerical pain rating scale, stand-reach test, and modified ODI, with a P < 0.05 in both groups after intervention
Alzahrani et al. (2021) Australia	26 participants were recruited from private physiotherapy practices in Sydney	12/14	IG: 49.0 ± 13.4 CG: 39.0 ± 13.8	Wearables-based walking intervention 8 weeks	Usual physiotherapy care 8 weeks	VAS ODI	Usual physiotherapy care plus a wearables-based walking intervention program was safe and moderately feasible and provided a significant reduction in pain.
Ahmad et al. (2023) India	31 patients, both men and women, with CLBP were recruited from December 2016 to April 2017	16/15	IG: 24.7 ± 5.56 CG: 25.9 ± 5.61	Retro-walking 5 weeks	Conventional treatment, three physiotherapy sessions per week for 3 weeks	NPRS ODI	Retro-walking provided an added advantage, as the experimental group showed a faster recovery, thus making it an effective treatment adjunct
Zuo et al. (2024) China	53 fighter pilots with chronic LBP	19/15	IG: 40.8 ± 8.1 CG: 36.6 ± 7.3	Core muscle exercise group five times/week for 12 weeks	IFC	ODI VAS	Combined therapy and core muscle exercise provided similar benefits in terms of core muscle function after 12 weeks of intervention therapy
Zou et al. (2019) China	43 Chinese community-dwellers were recruited in this study	15/15/13	IG: 58.13 ± 5.38 58.4 ± 5.08 CG: 60.67 ± 2.58	Tai chi chuan/core-stability training sessions thrice per week, with each session lasting 60 min for 12 weeks	Normal daily activities three sessions per week, with each session lasting 60 min for 12 weeks	VAS	Chen-style TCC and CST were found to have protective effects on NF in aging individuals with NLBP while alleviating non-specific chronic pain
Hall et al. (2011) Australia	160 subjects aged 18–70 years with persistent non-specific low-back pain volunteered to participate in the study	80/80	IG: 43.4 ± 13.5 CG: 44.3 ± 13.0	Tai chi 18 40-min sessions over a 10-week period	Usual healthcare	VAS RMDQ	A 10-week tai chi program improved pain and disability outcomes
Yan et al. (2022) China	Participants were recruited by an advanced community physician at three community universities	10/10	IG: 68.00 ± 1.15 CG: 70.00 ± 1.26	Tai chi 3 times a week for 6 weeks	Normal daily life.	VAS	6-week tai chi program can relieve pain and improve gait and dynamic balance in elderly women with CNSLBP
Cuesta-Vargas et al. (2011) America	Convenience sample of veterans with CLBP	23/23	IG: 37.6 ± 13.2 CG: 39.8 ± 11.2	MMPTP + DWR three times a week for 15 weeks	MMPTP three times a week for 15 weeks	VAS	Disability, health status, muscle strength and endurance, and lumbar range of motion significantly improved to a similar level in both intervention groups
Carvalho et al. (2020) Brazil	54 adult patients with CLBP were randomized either to an experimental or control group.=	27/27	IG: 47 ± 9.8 CG: 46 ± 10.9	AQE + DWR	No intervention	VAS RMDQ	Treatment with DWR was effective in the short term for achieving the desired outcome of pain reduction
Prado et al. (2021) Brazil	54 patients with CLBP were randomized to an experimental and a control group	27/27	IG: 35 ± 9.8 CG: 33 ± 11.3	Isostretching twice a week for 45 days	Waiting list for physical therapy	VAS RMDQ	Isostretching was effective in reducing p+B8:H34a and in improving function, patient satisfaction, and some aspects of quality of life
Turci et al. (2023) Brazil	Inclusion criteria: age 18–60 years, diagnosis of CNSLBP in the last 3 months	50/50	IG: 37 ± 13 CG: 37 ± 12	40-min stretch sessions, 8 weeks	Trunk stabilizing exercises 40-min sessions, 8 weeks	VAS	In people with CNSLBP, self-stretching exercises had very similar effects to motor-control exercises on pain intensity
Tekur et al. (2012) India	Assigned 80 (37 female and 43 male) patients with CLBP to yoga and physical exercise groups	40/40	IG: 49 ± 3.6 CG: 48 ± 4	Yoga 7 days	Physical exercise groups 7 days	VAS	7 day intensive residential yoga program reduced pain, anxiety, and depression and improved spinal mobility in patients with CLBP more effectively than physiotherapy exercises
Metri et al. (2023) India	Participants in this study were female teachers with CNSLBP working in secondary schools	20/18	IG: 39.8 ± 7.37 CG: 38.88 ± 6.67	Yoga 60-min 4 days/week for six consecutive weeks	No intervention	NPRS	A significant (p < 0.05) reduction in pain intensity and pain disability in the yoga group observed after 6 weeks
Ulger et al. (2023) Turkey	28 female patients included in the study	16 12	IG: 55.08 ± 2.67 CG: 47.12 ± 7.07	Yoga 2 days a week, 1 h each day for a total of 16 weeks	Stabilization exercise 2 days/week, 1 h each day for a total of 16 weeks	VAS ODI	Both exercise approaches were found to be similarly effective on pain, function, metabolic capacity, and sleep quality
Oz and Ulger (2024) Turkey	Participant eligibility criteria: aged 25–55 years	18/16	IG: 38 CG: 41	Yoga 6 weeks at a pace of three 60-min sessions per week	Physical therapy 6 weeks, at a pace of three 60-min sessions per week	VAS ODI	Uniquely focused solely on yoga as an intervention for non-specific CLBP
Tottoli et al. (2024) Brazil	145 individuals (aged 18–50 years) with LBP	72/73	IG: 35.7 ± 9 CG: 37.1 ± 9	Pilates twice a week, for 6 weeks	Home exercises twice a week for 6 weeks	NRS	Pilates was significantly superior to home exercise for pain and disability
Cruz-Díaz et al. (2017) Spain	Included patients with CLBP who responded to the recruitment advertisement through different health	34/30	IG 36.94 ± 12.46 CG 36.32 ± 10.67	Pilates 12 weeks		RMDQ VAS	Equipment-based and mat Pilates modalities are both effective in improving TaA (Transversus Abdominis Activation) activation in patients with CLBP
Miyamoto et al. (2018) Brazil	296 patients with CNSLBP	74/73	IG: 48.6 ± 15.8 CG: 47 ± 11.5	Pilates once a week for 6 weeks	Booklet	NRS RMDQ	Cost–utility analysis showed that Pilates three times a week was the preferred option
Batıbay et al. (2021) Turkey	60 female patients with CNSLBP	28/25	IG: 49.3 ± 10.4 CG: 48.4 ± 9.3	Pilates three times for 8 weeks	Home exercises thrice weekly for 8 weeks.	VAS ODI	Both Pilates and home exercises are effective in treating patients with CLBP
Asik and Sahbaz (2025) Turkey	64 participants with subacute LBP randomized into two groups	33/33		Pilates three times/week for 8 weeks	Home exercise three times/week for 8 weeks	VAS RMDQ	Pilates-based rehabilitation was more effective than home exercise program in improving pain, disability, and quality of life
Liu et al. (2019) China	43 individuals (aged 50 years or above) with CNSLBP	15/15/13	IG: 58.13 ± 5.38 58.4 ± 5.08 CG: 60.67 ± 2.58	Tai chi/core stability training three 60-min sessions per week for 12 weeks	Unaltered lifestyle	VAS	Tai chi and core stabilization training have significant effects on VAS for CNSLBP patients
Elabd and Elabd (2024) Egypt	50 CMLBP (22 male and 28 female) patients	25/25	IG: 33.04 ± 6.21 CG: 32.99 ± 5.98	Aerobic training program using a stationary bicycle for 8 weeks	Infrared, ultrasound, burst TENS, and exercises for 8 weeks	VAS ODI	Traditional program of infrared, ultrasound, TENS, and exercise is beneficial for CMLBP treatment
Williams et al. (2005) United States of America	Of the 60 subjects enrolled, 42 (70%) completed the study	22/20	IG: 48.7 ± 10.6 CG: 48.0 ± 1.96	Yoga 16 weeks		VAS	Preliminary data indicate that the majority of self-referred persons with mild CLBP will comply and report improvement on medical and functional pain-related outcomes from Iyengar yoga therapy
Nardin et al. (2022) Brazil	60 participants included in the survey (47 women and 13 men)	20/20	IG: 42.2 ± 9.1 CG: 43.1 ± 10.7	TGPBM twice a week for 4 weeks	GPBM twice a week for 4 weeks	VAS ODI	Effects of the combination of PBM and aquatic exercise have positive effects on reducing pain intensity, disability, and cortisol levels, but its effects on other variables (6WTA and CK) are too small to be considered significant
Yilmaz Yelvar et al. (2017) Turkey	Randomized controlled study conducted November 2014 to May 2015 in Turgut Ozal University Hospital	22/22	IG: 46.3 ± 3.4 CG: 52.8 ± 11.5	Virtual walking integrated physiotherapy five times a week for 2 weeks	Traditional physiotherapy five times a week for 2 weeks	VAS ODI	Virtual walking integrated physiotherapy reduces pain and kinesiophobia, improving function in patients with subacute and CNSLBP in the short term
Silva et al. (2018) Brazil	Study based on a randomized, controlled clinical trial involving 16 individuals	8 8	IG: 46.3 ± 3.4 CG: 47.00 ± 8.48	Pilates 12 sessions of 40 min	Conventional exercises Twelve sessions of 40 min	VAS ODI	Suggests that the method was effective for the group studied and proved suitable for the treatment of LBP, but it did not prove superior to conventional physical therapy
Miyamoto et al. (2013) Brazil	86 patients with CNSLBP.	43/43	IG: 40.7 ± 11.8 CG: 38.3 ± 11.4	Pilates 12 sessions, over 6 weeks	Booklet 12 sessions, over 6 weeks	NPRS RMDQ	Addition of modified Pilates exercises to an educational booklet provides small benefits compared with education alone in patients with CNSLBP; however, these effects were not sustained over time
Mazloum et al. (2018) Iran	47 patients with CNSLBP	16/16	IG: 37.1 ± 9.5 CG: 42.7 ± 8.1	Pilates over 6 weeks, 3 days per week	No interventions	VAS ODI	Estimated that core muscle activation and improving lumbopelvic rhythm in SP training may play a role in decreasing pain and physical disability in CLBP patients
Natour et al. (2015) Brazil	60 patients with CNSLBP diagnosis	30/30	IG: 48.08 ± 12.98 CG: 47.79 ± 11.47	Pilates	Medication treatment	VAS RMDQ	Pilates method can be used by patients + A1:H11 with LBP to improve pain, function, and aspects related to quality of life (functional capacity, pain, and vitality). Moreover, this method has no harmful effects on such patients
Nambi et al. (2014) India	60 subjects who fulfilled the selection criteria	30/30	IG: 44.26 ± 9.26 CG: 43.66 ± 8.82	Yoga 29 yogic postures training for 4 weeks	Strengthening 4 weeks	VAS	These results suggest that Iyengar yoga provides better improvement in pain reduction and improvement in HRQOL in CNSLBP than general exercise
Kuvačić et al. (2018) Croatia	30 individuals (age 34.2 ± 4.52 years) with CLBP	15/15	34.2 ± 4.52	Yoga 8-week (2 days per week)	Pamphlet	NRS ODI	Yoga program and education together appear to be effective in reducing depression and anxiety, which can affect perception of pain
Saper et al. (2017) United States	320 predominantly low-income, racially diverse adults with CNSLBP	127/64	IG: 46.4 ± 10.4 CG: 44.2 ± 10.8	Yoga 12 weekly yoga classes for 12 weeks	Educational book	VAS RMDQ	Manualized yoga program for CNSLBP was not inferior to PT for function and pain
Gladwell et al. (2006) United Kingdom	49 participants with CNSLBP for more than 12 weeks	20/14	IG: 36.9 ± 8.1 CG: 45.9 ± 8.0	Pilates 6 weeks	Normal activity	VAS	Pilates can improve general health, pain level, sports functioning, flexibility, and proprioception in individuals with CLBP
Neyaz et al. (2019) India	Patients between 18 and 55 years of age with complaint of CNSLBP persisting for 12 weeks	35/35	IG: 38 (26.5, 43) CG: 33 (27.5, 44)	Six standardized 35-min weekly Hatha yoga sessions	Conventional therapeutic exercises 35 mins per week sessions of CTEs	VAS RMDQ	Yoga provided similar improvement compared with CTEs in patients with CNSLBP
Cuesta-Vargas et al. (2012) Spain	70 potential patients	25/24	IG: 38.6 ± 12.2 CG: 37.8 ± 13.2	GP + DWR thrice weekly for 15 weeks	GP thrice weekly for 15 weeks	VAS RMDQ	For patients with CNSLBP, the addition of DWR to GP was more effective in reducing pain and disability than standard GP alone

VAS, visual analog scale; NPRS, numeric pain rating scale; ODI, Oswestry Disability Index; RMDQ, Roland–Morris Disability Questionnaire.

## Results

### Search results

A total of 8,445 potential studies were retrieved, with 1,505 duplicate studies excluded. After screening titles and abstracts, 48 studies were selected, of which 14 were excluded due to insufficient raw data and high risk of bias, leaving 35 studies eligible for inclusion (Table 1). The sample sizes of these 35 RCTs ranged from 8 to 127 participants. All 35 studies were published in English.

### Research characteristics

Interventions in the experimental groups were categorized into eight types: Pilates (Tottoli et al., 2024; Cruz-Díaz et al., 2017; Miyamoto et al., 2018; Batıbay et al., 2021; Asik and Sahbaz, 2025; Silva et al., 2018; Miyamoto et al., 2013; Mazloum et al., 2018; Natour et al., 2015; Gladwell et al., 2006) (n = 10), yoga (Tekur et al., 2012; Metri et al., 2023; Ulger et al., 2023; Oz and Ulger, 2024; Williams et al., 2005; Nambi et al., 2014; Kuvačić et al., 2018; Saper et al., 2017; Neyaz et al., 2019) ((n = 9), core-stability exercises (Zuo et al., 2024; Zou et al., 2019; Liu et al., 2019) (n = 3), tai chi (Zou et al., 2019; Liu et al., 2019; Hall et al., 2011; Yan et al., 2022) (n = 4), walking (Raza et al., 2023; Alzahrani et al., 2021; Ahmad et al., 2023; Yilmaz Yelvar et al., 2017) (n = 4), stretching (Prado et al., 2021; Turci et al., 2023) (n = 2), cycling (Elabd and Elabd, 2024) (n = 1), and deep-water running (Carvalho et al., 2020; Nardin et al., 2022; Cuesta-Vargas et al., 2011; Cuesta-Vargas et al., 2012) (n = 4).

### Risk of bias

**FIGURE 2:**
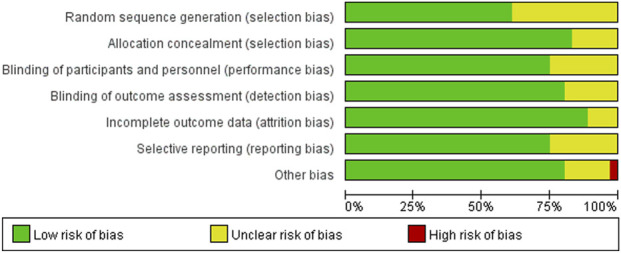
Risk-of-bias summary for the included studies (Cochrane).

**FIGURE 3:**
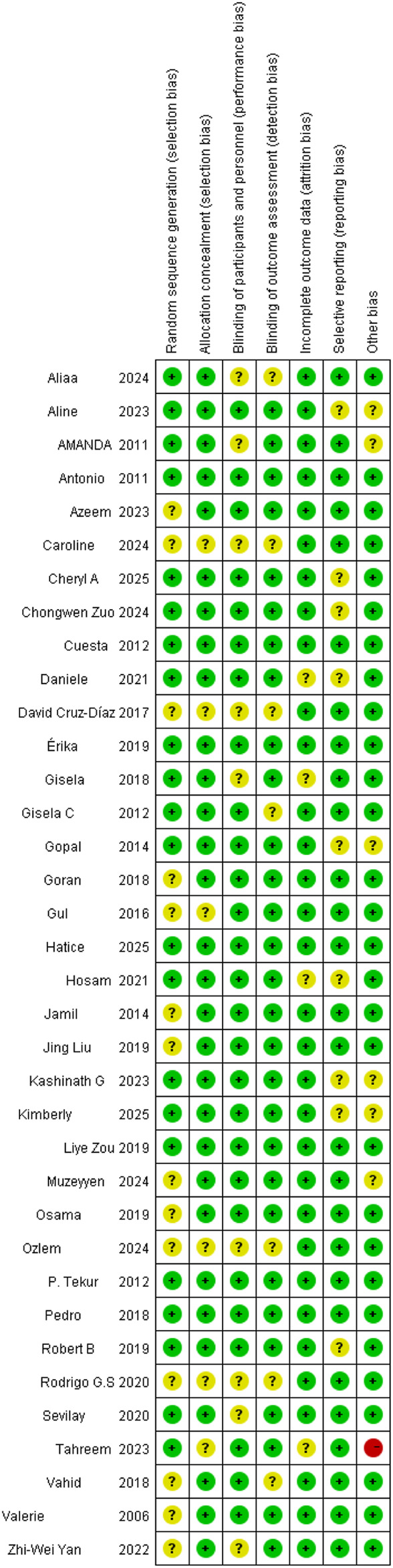
Detailed risk-of-bias assessments across individual domains of the Cochrane tool.

Based on the Cochrane Risk of Bias Assessment Tool, the studies included mostly showed a low risk of bias in terms of random sequence generation, allocation concealment, blinding of participants and personnel, blinding of outcome assessment, and incomplete outcome data. However, there was a certain degree of uncertainty regarding selective reporting and other biases. Specifically, most studies performed well in random sequence generation and allocation concealment, but uncertainties existed in selective reporting, which might affect the reliability of the study results. Therefore, during the meta-analysis, these potential biases required appropriate adjustment and interpretation. The results of the risk-of-bias assessment are presented in Figures 2, 3.

### Publication bias

**FIGURE 4:**
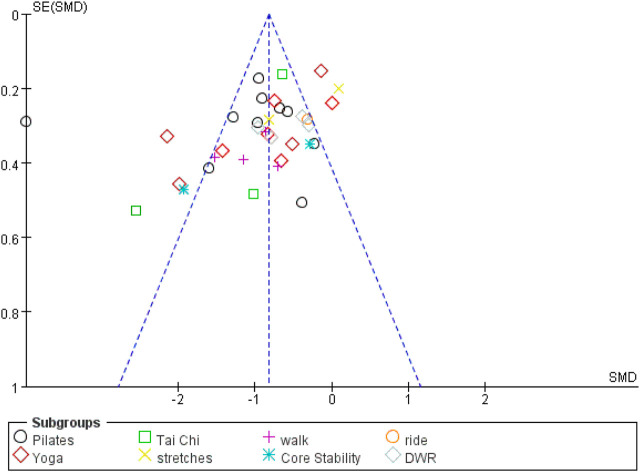
Funnel plot of all included studies.

As shown in Figure 4, the symmetrical funnel plot indicates no evidence of publication bias across the 35 included studies.

### Evidence quality

**FIGURE 5:**
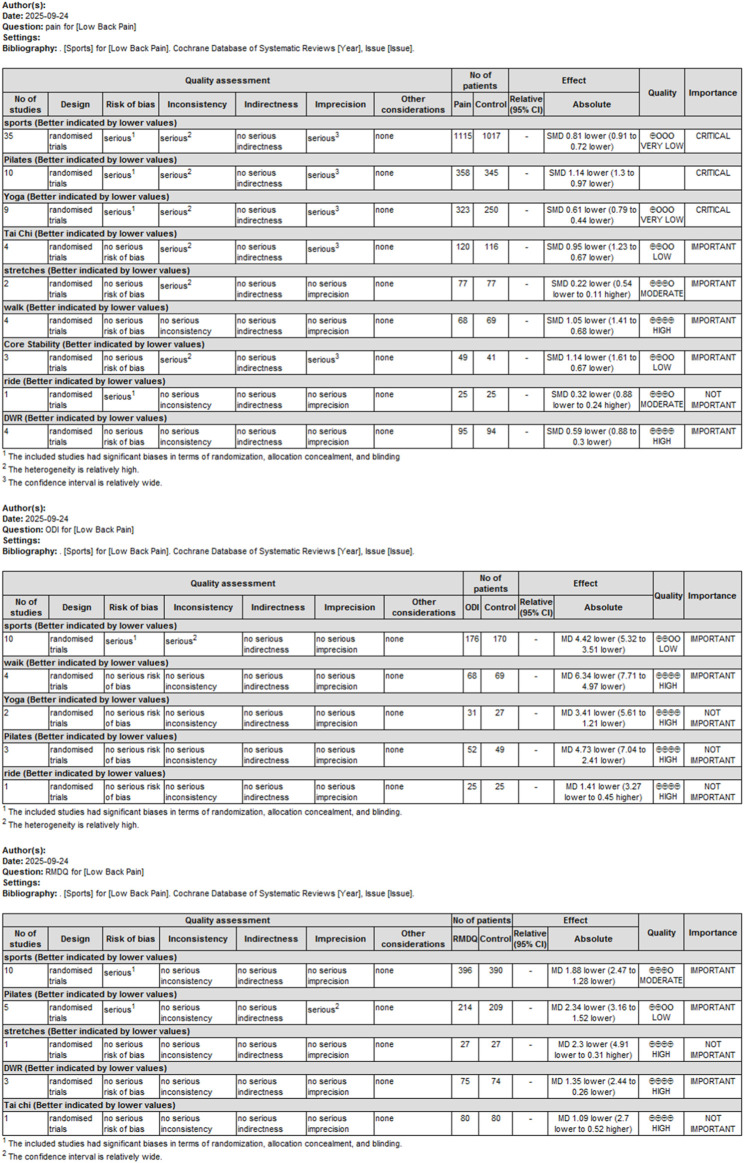
Summary of findings (SoF) table according to GRADE.

The quality of evidence for the primary outcome (pain) was assessed using the GRADE 4.0 approach. Initially, the evidence started at a high level since all included studies were randomized controlled trials. Subsequently, downgrading factors were examined item by item (Figure 5).

## Results of meta-analysis

### Pain

**FIGURE 6:**
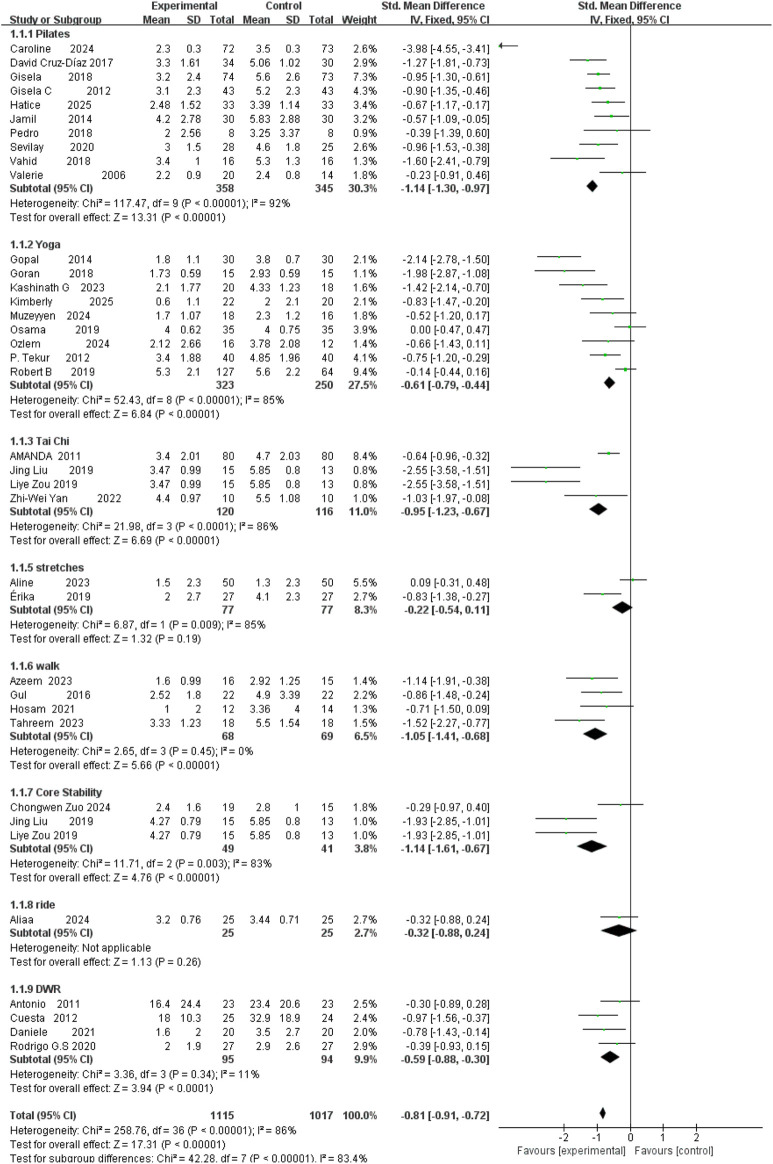
Forest plot of pain outcomes across different exercise interventions.

A total of 35 randomized controlled trials (RCTs), involving 2,132 participants (1,115 in intervention groups and 1,017 in control groups), were included in the meta-analysis. The overall pooled standardized mean difference (SMD) for pain relief was −0.81 (95% CI: −0.91 to −0.72), indicating a significant analgesic effect of exercise interventions (Z = 17.31, P < 0.001). However, substantial heterogeneity was observed across studies (I^2^ = 86%, P < 0.001). Pilates (SMD = −1.14; 95% CI −1.30, −0.97), yoga (SMD = −0.61; 95% CI –0.79 to −0.44), tai chi (SMD = −0.95; 95% CI –1.23 to −0.67), and walking (SMD = −1.05; 95% CI –1.41 to −0.68) all demonstrated clinically and statistically significant analgesic effects. Yoga and deep-water running (DWR) produced moderate but significant benefits, whereas stretching and cycling showed no significant effect. Notably, walking demonstrated zero heterogeneity, suggesting highly reproducible benefits. These findings support prioritizing Pilates, core-stability, walking, and tai chi in clinical or community-based exercise prescriptions (Figure 6).

### Oswestry Disability Index (ODI)

**FIGURE 7:**
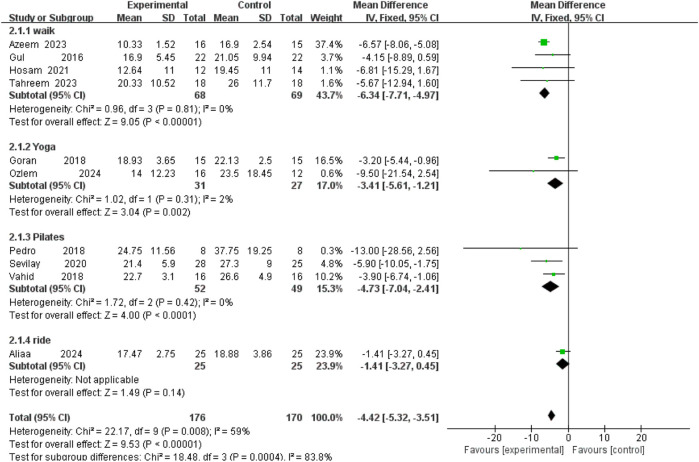
Forest plot of ODI outcomes across different exercise interventions.

Ten RCTs examined the effect of exercise on the ODI. The pooled estimate was 9.53 (346 participants) with moderate heterogeneity (P < 0.001; I^2^ = 59%). Subgroup analyses showed that walking, Pilates, and yoga all significantly reduced ODI scores compared with control. Walking demonstrated the largest effect (MD = –6.34; 95% CI –7.71 to–4.97; P < 0.001). Cycling did not significantly improve disability levels (Figure 7).

### Roland–Morris Disability Questionnaire (RMDQ)

**FIGURE 8:**
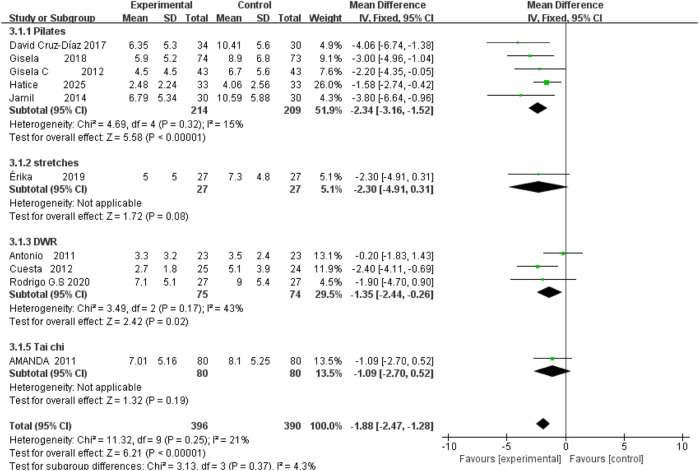
Forest plot of RMDQ outcomes across different exercise interventions.

Overall across 14 randomized trials (786 participants), exercise interventions outperformed control conditions (MD = −1.88, 95% CI –2.47 to −1.28, P < 0.001; I^2^ = 21%). Pilates yielded the largest and most consistent benefit (five studies, n = 423, MD = −2.34, 95% CI −3.16 to −1.52, I^2^ = 15%), whereas DWR produced a moderate effect (three studies, n = 149, MD = −1.35, 95% CI −2.44 to −0.26). Stretching and tai chi did not achieve statistical significance. These findings support prioritizing Pilates in exercise prescriptions for this outcome; evidence for other modalities remains inconclusive (Figure 8).

## Discussion

This study included 35 randomized controlled trials (n = 2,132), representing the first meta-analysis to compare the short-term efficacy of eight mainstream exercise programs on pain and functional impairment in chronic non-specific low-back pain (CNSLBP). The overall effect size SMD = −0.81 (95% CI −0.91 to −0.72) was not only statistically significant but also exceeded the MCID threshold based on VAS (Logroscino et al., 2005), indicating that the benefits of exercise intervention can be tangibly perceived by patients. At the subgroup level, walking, Pilates, and tai chi ranked the top three in analgesic effects. Notably, walking-related trials exhibited zero heterogeneity (I^2^ = 0%) and required minimal equipment, space, or specialized expertise, making it readily implementable in primary care, community rehabilitation, and even home settings. Functionally, walking yielded the greatest improvement in ODI (MD −6.34), while Pilates demonstrated the highest effect size on RMDQ (MD −2.34). Both exceeded their respective MCID thresholds (Atipas et al., 2022), indicating substantial relief from patients’ limitations in daily activities like lifting objects, prolonged standing, and bending. Although yoga reduced ODI scores, its lower confidence interval did not reach the MCID threshold, and its lack of procedural standardization suggests that it is better suited as an “enhancement module” within multimodal programs rather than a standalone core intervention. Stretching and cycling showed no clear benefits and should have their recommendation levels downgraded in clinical pathways. In summary, walking, Pilates, and tai chi can be considered first-line exercise prescriptions for LBP and are particularly suitable for resource-limited primary care settings or those requiring individualized rehabilitation.

Although all three top interventions fall under the category of low-impact aerobic exercise, their mechanisms for pain relief and functional recovery do not overlap. Walking induces rhythmic trunk sway, triggering alternating contractions of the lumbar multifidus and erector spinae muscles. This increases local blood flow shear stress, stimulating the release of beta-endorphins and serotonin. Simultaneously, periodic axial loading promotes intervertebral disc fluid exchange and reduces intrafibrous hydrostatic pressure, proving particularly effective for mechanically loaded pain (Ambrose and Golightly, 2015). Pilates emphasizes the triadic coordination of breathing–abdominal-pressure–pelvis, activating the transverse abdominis and diaphragm within a closed kinetic chain to create a pneumatic lumbar-support effect that instantly reduces segmental misalignment. Its movement sequences primarily focus on sagittal plane control, making it most suitable for improving the “lifting objects” and “prolonged standing” items in the ODI (Huxel Bliven and Anderson, 2013). Tai chi combines slow eccentric contractions with focused attention. fMRI studies confirm that it downregulates excitability in the insula-thalamic pain network, offering central analgesia benefits for patients with anxiety or catastrophic thinking (Srivatsa et al., 2018). Therefore, bedside decisions may rapidly triage patients based on pain phenotypes: walking is preferred for those with excessive mechanical load; Pilates is prioritized for segmental instability or early postoperative cases; tai chi is added for those with emotional distress or high fall risk.

Admittedly, the present meta-analysis is constrained by substantial heterogeneity (I^2^ > 85%) that permeates both the overall pool and the Pilates/yoga strata. This dispersion is neither stochastic nor purely methodological; rather, it stems from three converging layers. At the patient-level, discogenic, facet-joint, and sacroiliac subtypes display up to two-fold differences in segmental stiffness under combined shear–torsional loading, and such mechanical heterogeneity is known to modulate exercise responsiveness independently of symptom duration or body mass index (BMI) (Bisschop et al., 2013). At the trial-level, dosage descriptors (frequency, session length, and axial-load progression) were reported inconsistently, while the interchangeable use of VAS and NRS without study-specific conversion inflated the residual variance by approximately 8%–12%. At the evidence-level, 40% of eligible trials were not pre-registered, and the selective publication of positive findings shifted the pooled mean upward, widening the 95% prediction interval (Hohlfeld et al., 2024). Consequently, the summary effect should be interpreted as an upper-bound estimate of real-world benefit rather than a single “true” value. To mitigate this uncertainty, we recommend initiating a multi-center IPD consortium that integrates three-dimensional data from imaging, biomechanics, and psychology to construct a “pain phenotype-exercise prescription” predictive model. In addition, we recommend conducting a pragmatic stepped-wedge RCT to validate the cost-effectiveness of the two-stage “walking + Pilates” intervention at the community primary care level. Simultaneously, wearable sensors will monitor trunk-tilt angle, step frequency, and electromyography in real time to develop an AI-driven remote supervision platform to enable precise exercise dose titration. The ultimate goal is to advance exercise intervention from “experience-based exercise selection” to “data-driven dose determination,” providing an affordable, sustainable, and replicable precision rehabilitation pathway for LBP (Ganesh et al., 2023).

## Conclusion

Building on our results, clinicians should match exercise to the patient with chronic low-back pain: brisk, equipment-free walking—uniformly effective and well tolerated—suits older or deconditioned adults whose pain and disability are most severe; augmenting walking with Pilates best supports those in the chronic stage who need greater core-stability and relapse prevention; tai chi, when supervised, provides a mind–body adjunct for well-coordinated individuals seeking additional analgesic and functional gains. However, many modalities rest on small, short-term trials, dose–response relationships remain undefined, and the influence of pain phenotypes and comorbidities is unknown. Future, large, high-quality randomized control trials should therefore validate understudied options such as cycling, delineate minimal effective and maximal tolerable doses through dose–response modeling, extend follow-up to capture recurrence and quality-of-life trajectories, and integrate imaging with biomechanical markers to clarify mechanisms to advance precision rehabilitation.

## Data Availability

The original contributions presented in the study are included in the article/supplementary material; further inquiries can be directed to the corresponding author.
